# Bis(formylphenolato)cobalt(II)-Mediated Alternating Radical Copolymerization of *tert*-Butyl 2-Trifluoromethylacrylate with Vinyl Acetate

**DOI:** 10.3390/polym9120702

**Published:** 2017-12-12

**Authors:** Sanjib Banerjee, Ekaterina V. Bellan, Florence Gayet, Antoine Debuigne, Christophe Detrembleur, Rinaldo Poli, Bruno Améduri, Vincent Ladmiral

**Affiliations:** 1 Institut Charles Gerhardt Montpellier, University of Montpellier, CNRS, ENSCM, Place Eugène Bataillon, 34095 Montpellier CEDEX 5, France; sanjib.banerjee@iitbhilai.ac.in (S.B.); bruno.ameduri@enscm.fr (B.A.); 2 Laboratoire de Chimie de Coordination (LCC), Université de Toulouse, CNRS, UPS, INPT, 205 route de Narbonne, BP 44099, 31077 Toulouse CEDEX 4, France; ekaterina.bellan@gmail.com (E.V.B.); florence.gayet@lcc-toulouse.fr (F.G.); 3 Center for Education and Research on Macromolecules (CERM), University of Liege, CESAM Research Unit, Sart-Tilman B6a, 4000 Liege, Belgium; adebuigne@ulg.ac.be (A.D.); christophe.detrembleur@ulg.ac.be (C.D.); 4 Institut Universitaire de France, 1, rue Descartes, 75231 Paris CEDEX 05, France

**Keywords:** alternating copolymers, cobalt complex, fluoropolymer, organometallic mediated radical polymerization, poly(vinyl acetate)

## Abstract

The organometallic-mediated radical polymerization (OMRP) of vinyl acetate (VAc) and its OMR copolymerization (OMRcoP) with *tert*-butyl 2-trifluoromethylacrylate (MAF-TBE) mediated by Co(SAL)_2_ (SAL = 2-formylphenolato or deprotonated salicylaldehyde) produced relatively well-defined PVAc and poly(VAc-*alt*-MAF-TBE) copolymers at moderate temperature (<40 °C) in bulk. The resulting alternating copolymer was characterized by ^1^H-, ^13^C- and ^19^F-nuclear magnetic resonance (NMR) spectroscopies, and by size exclusion chromatography. The linear first-order kinetic plot, the linear evolutions of the molar mass with total monomer conversion, and the relatively low dispersity (*Đ*~1.55) of the resulting copolymers suggest that this cobalt complex provides some degree of control over the copolymerization of VAc and MAF-TBE. Compared to the previously investigated cobalt complex OMRP mediators having a fully oxygen-based first coordination sphere, this study emphasizes a few peculiarities of Co(SAL)_2_: a lower ability to trap radical chains as compared to Co(acac)_2_ and the absence of catalytic chain transfer reactions, which dominates polymerizations carried in the presence of 9-oxyphenalenone cobalt derivative.

## 1. Introduction

**Scheme 1:**
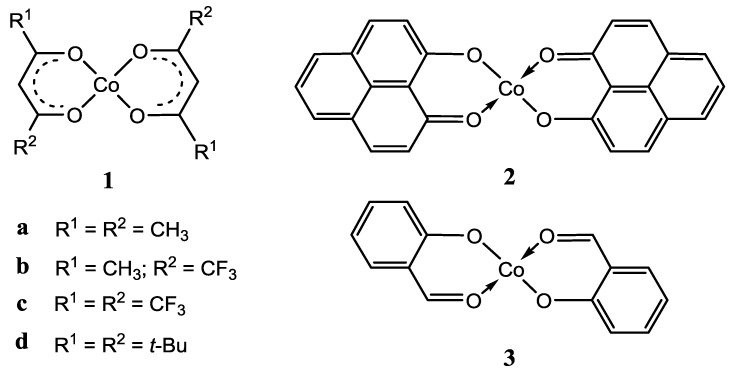
Schematic representations of the cobalt(II) complexes: bis(acetylacetonato)cobalt(II) (**1a**); bis (1,1,1-trifluoro-acetylacetonato)cobalt(II) (**1b**); bis(1,1,1,5,5,5-hexafluoropentan-2,4-dionato)-cobalt(II) (**1c**); bis(2,2,6,6-tetramethylheptan-3,5-dionato)cobalt(II) (**1d**); bis(9-oxyphenalenonato)-cobalt(II) (**2**), and; bis(2-formylphenolato)cobalt(II) (**3**).

Fluoropolymers are unique materials, exhibiting outstanding properties, suitable for high-value added applications [1,2]. The incorporation of sequences of these polymers into a di- or multi-block [3] polymer architectures could lead to interesting materials of even greater value. Extensive research and development of different reversible deactivation radical polymerization (RDRP) techniques over the last two decades has enabled the facile synthesis of macromolecules with a high degree of complexity and well-defined architectures [4,5]. Although nitroxide-mediated radical polymerization (NMP) [6], atom transfer radical polymerization (ATRP) [7], iodine-transfer polymerization (ITP) [8,9,10] and reversible addition-fragmentation chain transfer (RAFT) polymerization [11,12,13,14,15] have been comparatively more studied, organometallic-mediated radical polymerization (OMRP) [16,17,18,19,20] has recently emerged as a potent RDRP technique for the polymerization of “less activated monomers” (LAMs), such as vinyl imidazolium salts [21,22] or vinyl amides (such as vinyl pyrrolidone) [23]. Indeed, for these more difficult monomers, OMRP and, in particular, cobalt-mediated radical polymerization (CMRP) [24] has proven very efficient, opening synthetic routes for homopolymers and block copolymers that could only be prepared with a lower level of control or not at all by other RDRP techniques. The technique was pioneered by Wayland et al. [25] using tetramesitylporphyrin for the controlled polymerization of acrylates, and since then several other Co^II^ complexes with different donor atom combinations and coordination geometries have been reported. However, the controlled polymerization of LAMs seems so far restricted to complexes supported by chelating oxygen-based ligands, like the archetypic bis(acetylacetonate)cobalt(II) complex, Co(acac)_2_ (**1a**), first introduced by Debuigne et al. [26] for the controlled polymerization of vinyl acetate (VAc). To the best of our knowledge, investigations of other Co^II^ complexes entirely supported by oxygen atoms in OMRP has, so far, been limited to the 1,1,1-trifluoro- and 1,1,1,5,5,5-hexafluoropentan-2,4-dionates (**1b** and **1c**) [27], to the 2,2,6,6-tetramethylheptan-3,5-dionate (**1d**) [28] and to the 9-oxyphenalenone derivative (**2**) [29] (Scheme 1).

We are interested in expanding the available array of cobalt(II) complexes as OMRP mediators and to understand how the metal coordination sphere affects the metal controlling ability, potentially providing access to the controlled polymerization of new challenging monomers such as the fluorinated olefins [30,31]. The focus of the present article is the assessment, for the first time, of the air-stable bis(2-formylphenolato)cobalt(II) complex (Co(SAL)_2_, **3**, Scheme 1), where the 2-formylphenolate is the deprotonated form of salicylaldehyde. This complex has already been described in the literature [32,33] and is quite easily synthesized. Its performances in the bulk polymerization of VAc and in the bulk copolymerization of VAc and *tert*-butyl 2-trifluoromethylacrylate (MAF-TBE) are evaluated here. The combination of these two monomers was recently shown to radically polymerize in a strictly alternating fashion (their reactivity ratios are essentially zero) [34] and to be well controlled by Co(acac)_2_ [35]. Fluorinated copolymers containing 2-(trifluoromethyl)acrylic acid (MAF) and alkyl 2-trifluoromethacrylates (MAF-esters) may have applications in molecularly imprinted polymers, microlithography, polymer gel electrolyte membranes for fuel cells, and polymer electrolytes for Li-ion batteries [36]. Such copolymers may also contribute to the development of new materials with enhanced properties (e.g., high hydrophobicity, adhesion) [37,38,39] for high-tech applications (e.g., functional coatings or membranes).

## 2. Materials and Methods

### 2.1. Materials

Vinyl acetate (VAc, ≥99%, Sigma-Aldrich Chimie, Saint Quentin-Fallavier, France) and *tert*-butyl 2-trifluoromethylacrylate (MAF-TBE, kindly donated by Tosoh F-Tech Company, Shunan, Japan) were stored under nitrogen and purged for 30 min with nitrogen before use. 2,2,6,6-Tetramethyl-1-piperidinyloxy (TEMPO) (Acros Organics, Geel, Belgium), mesitylene (Mes) (>99.9%, Sigma-Aldrich Chimie, Saint Quentin-Fallavier, France), and 2,2′-azobis(4-methoxy-2,4-dimethylvaleronitrile) (V-70, Wako chemicals, Neuss, Germany) were used as received. Acetone and laboratory reagent grade pentane (purity > 95%) were purchased from Sigma-Aldrich and used as received. The deuterated solvents used for nuclear magnetic resonance (NMR) spectroscopy THF-*d*_8_, acetone-*d*_6_ and CDCl_3_ were purchased from Euroiso-top (Grenoble, France) (purity > 99.8%). The cobalt complex **3** was prepared according to the published procedure [32,33].

### 2.2. Experimental Procedures

#### 2.2.1. OMRP of VAc Initiated by V-70 in the Presence of **3**

All manipulations were carried out under a protective argon atmosphere. Compound **3** (0.087 mmol, 0.026 g) and V70 (0.174 mmol, 0.054 g) were introduced into the Schlenk tube and purged by three vacuum-argon cycles, followed by the addition of degassed vinyl acetate (4 mL, 43.4 mmol) and 0.2 mL of mesitylene. The reaction mixture was degassed by two freeze/pump/thaw cycles. Then, V-70 was added to the reaction mixture and the Schlenk tube was immersed in an oil bath at 30 °C under magnetic stirring. Aliquots were withdrawn periodically to monitor the reaction progress by ^1^H-NMR and size exclusion chromatography (SEC). The monomer conversion was determined by ^1^H-NMR spectroscopy in THF-*d*_8_ using mesitylene as an internal standard. The samples for SEC characterization were quenched with excess TEMPO, then precipitated from pentane and dried under vacuum. The VAc conversion was calculated using Equation (1) or (2) where $\int_{i}^{j}\mathrm{CH}$ stands for the integral of the CH signal ranging from i ppm to j ppm in the ^1^H-NMR spectrum at time *t* (unless stated otherwise).

(1) $$\%\mathrm{VAc}\;\mathrm{conversion}=\frac{\left(\int_{4.75}^{5.20}\mathrm{CH}-\int_{4.35}^{4.65}\mathrm{CH}\right)}{\int_{4.75}^{5.20}\mathrm{CH}}\times 100$$

(2) $$\%\mathrm{VAc}\;\mathrm{conversion}=\frac{\left[\int_{7.10}^{7.40}\mathrm{CH}\left(\mathrm{VAc}\right)/\int_{6.60}^{6.90}\mathrm{CH}\left(\mathrm{mesitylene}\right)\right]}{{\left[\int_{7.10}^{7.40}\mathrm{CH}\left(\mathrm{VAc}\right)/\int_{6.60}^{6.90}\mathrm{CH}\left(\mathrm{mesitylene}\right)\right]}_{t=0}}\times 100$$

#### 2.2.2. Co(SAL)_2_-Mediated Radical Copolymerization of VAc and MAF-TBE

All copolymerizations were performed under a dry dinitrogen atmosphere using Schlenk techniques. In a typical copolymerization, V-70 (278 mg, 0.9 mmol) and **3** (54 mg, 0.3 mmol) were placed into a Schlenk flask and purged by three vacuum-nitrogen cycles. Then, the degassed monomers, VAc (7.4 mL, 80.5 mmol) and MAF-TBE (14.1 mL, 80.5 mmol) were introduced into the flask under a nitrogen flux and the reaction mixture was heated at 40 °C under magnetic stirring. During polymerization, samples were withdrawn from the reaction medium at regular intervals to monitor the monomer conversions by ^1^H-NMR spectroscopy (for VAc) and ^19^F-NMR spectroscopy (for MAF-TBE), and for polymer molar masses and dispersities (*Đ*) determinations using SEC (see SI, Table S2, Figures S1 and S2). All the samples were quenched with an excess of TEMPO (solution in degassed THF, typically 6 equivalents with respect to the number of moles of **3** used in the copolymerization) to remove the residual cobalt complex from the polymer chain-end before the SEC analysis. The monomer conversions were determined by ^1^H-NMR (for VAc) and ^19^F-NMR (for MAF-TBE) spectroscopies using Equations (3) and (4), respectively, where $\int_{i}^{j}\mathrm{CH}$ and $\int_{n}^{m}{\mathrm{CF}}_{3}$ stand for the integral of the CH signal, ranging from i ppm to j ppm in the ^1^H-NMR spectrum at time *t*, and that of the CF_3_ signal, ranging from *n* ppm to *m* ppm in the ^19^F-NMR spectrum at time *t*.

(3) $$\%\mathrm{VAc}\;\mathrm{conversion}=\frac{\int_{5.00}^{5.40}\mathrm{CH}}{\left(\int_{4.35}^{4.65}\mathrm{CH}+\int_{5.00}^{5.40}\mathrm{CH}\right)}\times 100$$

(4) $$\%\;\mathrm{MAF}-\mathrm{TBE}\;\mathrm{conversion}=\frac{\int_{-68.5}^{-70.5}{\mathrm{CF}}_{3}}{\int_{-66.5}^{-67.5}{\mathrm{CF}}_{3}+\int_{-68.5}^{-70.5}{\mathrm{CF}}_{3}}\times 100$$

After completion of the reaction, the unreacted monomers were removed under vacuum. The remaining crude product was dissolved in acetone and precipitated twice from chilled pentane. It was then filtered through a filter funnel, and dried under vacuum (10^−3^ bar, 40 °C) for 12 h. The purified copolymers were characterized by ^1^H-, ^19^F- and ^13^C-NMR spectroscopies (see Supporting Information, Figures S3–S5) and by SEC (see Section 3).

### 2.3. Characterizations

#### 2.3.1. Size Exclusion Chromatography (SEC)

The molar masses (*M_n_*s) and dispersities (*Ð*s) of the polymers were assessed by size exclusion chromatography (SEC). The SEC system (Agilent Technologies, Les Ulis, France) used for the poly(VAc-*alt*-MAF-TBE) copolymer analysis was equipped with a PL0390-0605390 LC light scattering detector functioning at two diffusion angles (15° and 90°), a PL0390-06034 capillary viscometer, and a 390-LC PL0390-0601 refractive index detector and two PL1113-6300 ResiPore 300 × 7.5 mm columns. The entire SEC system was equilibrated at 35 °C. DMF (containing 0.1 wt % of LiCl), at a flow rate of 0.8 mL min^−1^ was used as the eluent, while toluene was used as the flow rate marker. Poly(methyl methacrylate) standards were used for calibration and typical sample concentration employed was 10 mg mL^−1^. The results were processed using the corresponding Agilent software. The SEC analyses of the poly(vinyl acetate) samples were carried out in filtered THF (flow rate: 1 mL min^−1^) at 35 °C on a 300 × 7.5 mm PL gel 5 µm mixed-D column (Polymer laboratories) equipped with multi-angle light-scattering (Mini Dawn Wyatt) and refractive index (RI2000, Sopares or Wyatt Optilab Rex) detectors. The results were processed using Astra 6 software, using a *dn*/*dc* value of 0.085.

#### 2.3.2. Nuclear Magnetic Resonance (NMR) Spectroscopy

The ^1^H-, ^13^C- and ^19^F-NMR spectra were recorded on a Bruker AC 400 Spectrometer (400 MHz for ^1^H, 100 MHz for ^13^C and 376 MHz for ^19^F) using CDCl_3_, THF-*d*_8_ or acetone-*d*_6_ as a solvent using the following experimental conditions for ^1^H- (or ^13^C- or ^19^F)-NMR spectra: flip angle 90° (or 90°or 30°); acquisition time 4.5 s (or 0.3 s or 0.7 s); pulse delay 2 s (or 1 or 5 s); number of scans 32 (or 8192 or 64), and; a pulse width of 12.5, 9.5 and 5.0 μs for ^1^H-, ^13^C- and ^19^F-NMR, respectively. Coupling constants and chemical shifts are presented in Hertz (Hz) and parts per million (ppm), respectively. ^1^H decoupling was performed with waltz16. ^19^F decoupling was performed with nested loops using 0.5 ms and 1 ms chirped adiabatic pulses with 80 kHz band with in order to desynchronize and minimize decoupling artifacts.

## 3. Results and Discussion

**Scheme 2:**
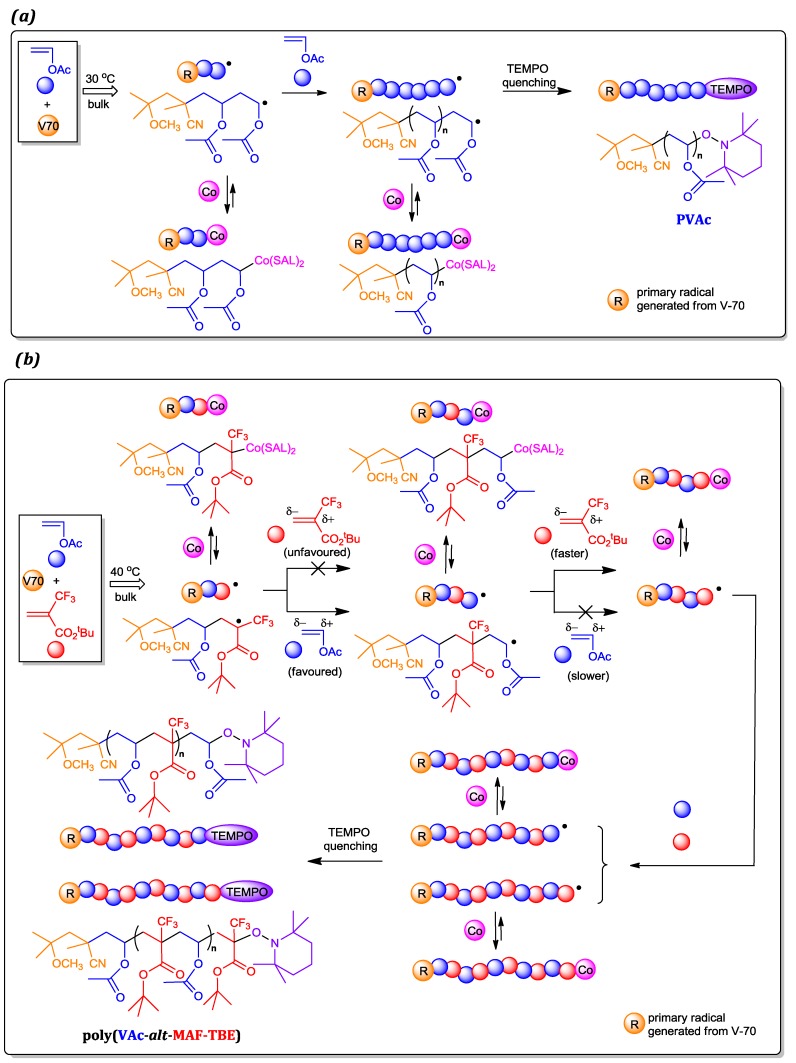
Schematic representations of the cobalt-mediated radical polymerization (CMRP) of vinyl acetate (VAc) (**a**) and of VAc and *tert*-butyl 2-trifluoromethylacrylate (MAF-TBE) (**b**) mediated by **3**. In (**b**), the first addition of the primary radical (R) to VAc is arbitrarily assumed. The alternative R addition to MAF-TBE is also possible.

The performance of **3** as an OMRP mediator was assessed in the homopolymerization of VAc (Scheme 2a) and in the alternating copolymerization of VAc and MAF-TBE (Scheme 2b).

### 3.1. Homopolymerizations of VAc and MAF-TBE Mediated by ***3***

**Figure 1:**
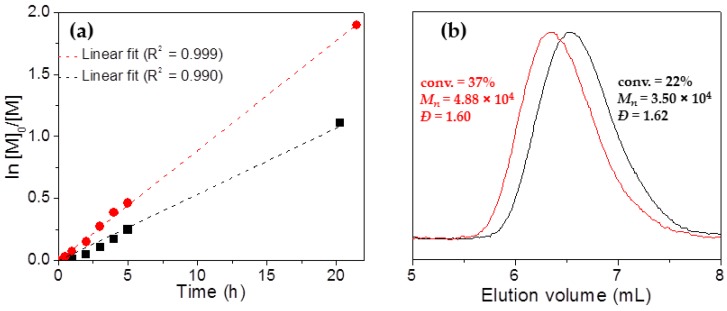
First order kinetic plot (**a**) and representative size exclusion chromatography (SEC) traces (**b**) for the radical polymerization of VAc mediated by **3** in bulk at 30 °C initiated by V-70 in the presence {[VAc]_0_/[V-70]_0_/[3]_0_ = 500/2/1, (■)} and absence {[VAc]_0_/[V-70]_0_ = 500/2, (●)}, of **3**.

Bulk homopolymerizations of VAc were carried out at 30 °C using V-70 as initiator in the presence and absence of **3**. A V-70/**3** ratio of 2 was used in the cobalt-mediated process, namely, conditions that allow a degenerate transfer controlling mechanism (excess radicals with respect to the putative organometallic dormant species). A first important observation is the absence of an induction time. This contrasts with the behavior reported for the same polymerization controlled by **1a** [26,40], **1b** [27] and **1d** [28], where essentially no polymer was formed until all the Co^II^ complex was converted into the organometallic dormant species PVAc-Co^III^, followed by controlled polymerization by degenerative transfer. On the other hand, the polymerization carried out in the presence of complex **2** [29] also exhibited no induction time, as in the present case. The conventional radical polymerization (no cobalt complex) ran slightly faster than the polymerization mediated by **3**, with apparent first-order *k_p_* values of 2.5 × 10^−5^ s^−1^ (in the absence of complex) and *k_p_ =* 1.5 × 10^−5^ s^−1^ (with **3**), see Figure 1a. Although the polymer dispersities (*Đ*) were similar at relatively low conversion (1.62 at 22% in the presence of **3** and 1.60 at 37% in the conventional radical polymerization, Figure 1b and Table S1), the dispersity increased to 1.99 at higher conversions for the PVAc obtained by conventional radical polymerization and only to 1.66 for that of the polymerization mediated by **3**. The polymerization carried out in the presence of **2**, using the same VAc/V-70/Co ratio and temperature, gave a significantly lower apparent *k_p_* of 2.0 × 10^−6^ s^−1^ [29]. These results suggest that **3** has the ability to reversibly interact with the growing radical chains, though less than **2** and much less than **1a**, **1c** and **1d**, and that the trapping equilibrium is not sufficiently displaced in favor of the dormant species to ensure a good level of control. However, the low solubility of **3** in the VAc monomer may also contribute to this poor control. It is also relevant to note that there is no evidence of catalytic chain transfer (CCT), contrary to the polymerization mediated by **2**. Indeed, the isolated polymers had higher molar masses, according to the SEC analyses (Table S1), than predicted for a controlled polymerization. Attempts to obtain a homopolymer from MAF-TBE in the presence of **3** failed as expected, since this monomer does not homopolymerize under radical conditions [35,36].

### 3.2. Copolymerization of VAc and MAF-TBE Mediated by ***3***

**Figure 2:**
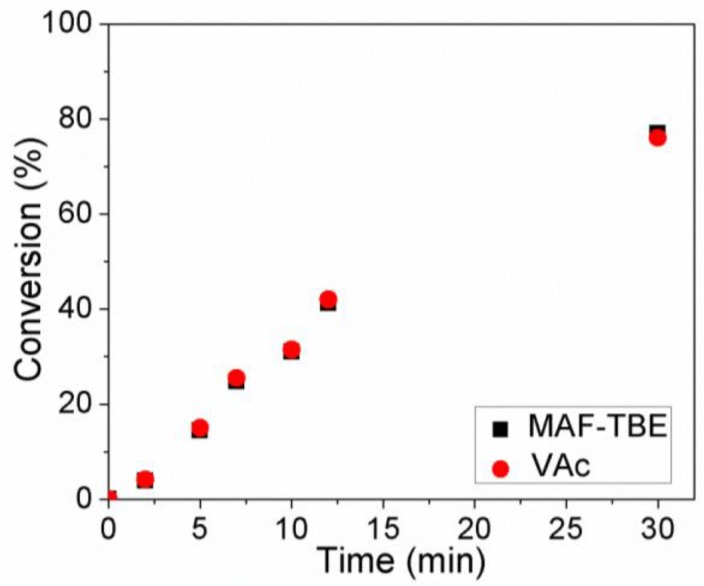
Conversion of VAc and MAF-TBE vs. time for the bulk copolymerization of VAc and MAF-TBE at 40 °C initiated by V-70 in the presence of **3** {[VAc]_0_ + [MAF-TBE]_0_}/[V-70]_0_/[3]_0_ = 537/3/1, [VAc]_0_/[MAF-TBE]_0_ = 1/1.

Following this initial result with VAc, the bulk copolymerization of VAc and MAF-TBE (1:1 feed) mediated by **3** was investigated (Scheme 2b, Table S2). First, it should be pointed out that **3** exhibits greater solubility in the VAc/MAF-TBE mixture than in neat VAc (and even greater in neat MAF-TBE), although it was not fully soluble in the initial stages of the polymerization. The solutions became homogeneous at higher conversions as all the complex became incorporated in the propagating polymer chains. The copolymerization progress was monitored by ^1^H- and ^19^F-NMR spectroscopies to determine the VAc and MAF-TBE conversions, respectively (the stacked spectra are shown in Figures S1 and S2). As expected, since these monomers are known to copolymerize in an alternating manner [34,35], equimolar conversions of VAc and MAF-TBE were recorded throughout the polymerization (Figure 2 and Table S2). The broad ^1^H NMR signal centered at 5.20 ppm (Figure S1) was assigned to the methine group of VAc in the VAc-MAF-TBE alternating dyad [35]. In contrast, the methine group in the PVAc homopolymer gives a signal at 4.80 ppm [41].

Confirmation of the alternating structure was supported by detailed ^1^H-, ^19^F- and ^13^C-NMR characterizations (Figures S3–S5), in excellent agreement with the previously published spectroscopic characterization of this polymer [34,35]. Notably, the ^1^H-NMR spectrum (Figure S3) shows the characteristic resonance of the VAc –CHOAc units in the VAc-MAF-TBE alternating dyad [34,35,42,43,44,45], which is significantly shifted from that of the same units in the homopolymer, and those of the –C(CH_3_)_3_ of MAF-TBE units [34,35]. The notable absence of –CHOAc signal corresponding to the PVAc homopolymer at 4.8 ppm [41] is consistent with the exclusive formation of an alternating copolymer. The ^19^F-NMR spectrum (Figure S4) reveals the characteristic signal of the –CF_3_ group in the MAF-TBE units at −69 ppm [46]. The ^13^C{^19^F}-NMR spectrum (Figure S5) allows the full resonance assignment in accordance to the expected poly(VAc-*alt*-MAF-TBE) structure [35]. The resonance centered at 67.5 ppm has a complex shape, probably because of the presence of different stereoisomers in the alternating MAF-TBE-VAc-MAF-TBE triads.

**Figure 3:**
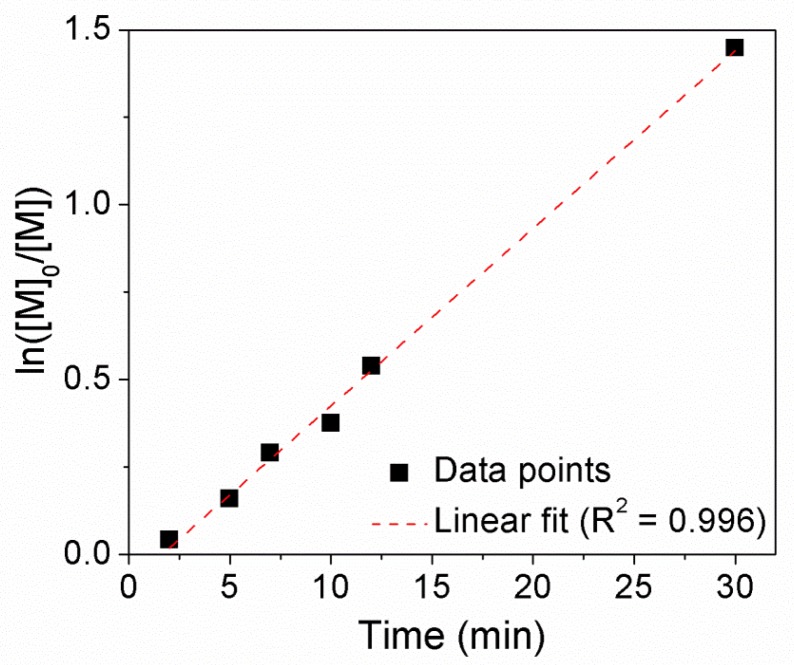
First order plot for the bulk copolymerization of VAc and MAF-TBE at 40 °C initiated by V-70 in the presence of **3** {[VAc]_0_ + [MAF-TBE]_0_}/[V-70]_0_/[3]_0_ = 537/3/1, [VAc]_0_/[MAF-TBE]_0_ = 1/1.

The first-order kinetic plot of this copolymerization (Figure 3) is linear, consistent with a constant radical concentration throughout the copolymerization. In addition, an induction period of about 1.5 h was observed, consistent with the higher initial solubility of **3** in the VAc/MAF-TBE mixture and confirming that **3** is indeed able to trap all radicals until the polymerization kicks off by degenerate transfer once all **3** is converted to the dormant alkylcobalt(III) complex.

**Figure 4:**
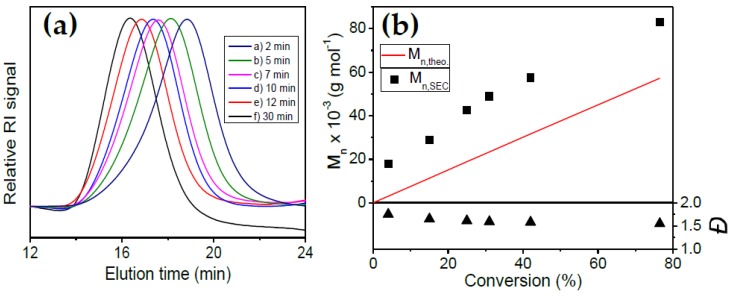
Evolutions of the SEC traces vs. elution time (**a**) and dependence of *M_n_* and *Đ* vs. total monomer conversion (**b**) for the bulk copolymerization of VAc with MAF-TBE at 40 °C initiated by V-70 in the presence of **3**, {[VAc]_0_ + [MAF-TBE]_0_}/[V-70]_0_/[3]_0_ = 537/3/1, [VAc]_0_/[MAF-TBE]_0_ = 1/1. The straight line in (**b**) represents the theoretical *M_n_*, based on one chain per Co atom.

The SEC traces of this alternating copolymerization (Figure 4a) remained monomodal throughout the reaction, with an essentially linear evolution of the copolymer molar mass (*M_n_*) versus monomer conversion (Figure 4b) and dispersities that decreased as conversion increased. These are characteristic features of a controlled polymerization. However, the dispersity remained relatively high (*Đ* = 1.55–1.75) compared with that of the corresponding Co(acac)_2_-mediated copolymerization [35]. The initial (<20% monomer conversion) poorly controlled phase, indicated by the higher *Đ* values is probably partially due to the incomplete solubility of **3** in the bulk monomer mixtures.

## 4. Conclusions

In conclusion, this study reports, for the first time, the action of bis(2-formylphenolato)cobalt(II) complex as an OMRP controlling agent. The moderating equilibrium is less in favor of the dormant species relative to species **1a**, **1b** and **1d**, not allowing a sufficient level of control for the homopolymerization of VAc. This effect is similar to that recently reported for the 9-oxyphenalenone derivative (2) [29], which has a similar structure to complex **3**. However, contrary to the VAc polymerization mediated by **2**, the one mediated by **3** shows no evidence of catalytic chain transfer to monomer, indicating that **3** not only makes homolytically weaker PVAc-Co^III^ bonds in the dormant species relative to **1a**, but also has a lower aptitude to abstract a *β*-H atom from the growing radical PVAc chains. The OMRP testing of **3** was then extended to the alternating copolymerization of VAc and MAF-TBE, enabling a relatively well-controlled synthesis of a strictly alternating poly(VAc-*alt*-MAF-TBE) copolymer under degenerative transfer conditions. The moderately controlled behavior of this alternating copolymerization is suggested by the linear semilogarithmic plot, the linear *M_n_*-conversion plot, and the acceptable *Đ* values which decrease as the conversion increases. The alternating structure of the copolymer was confirmed by detailed microstructure analysis using NMR.

We have therefore extended the array of available Co^II^ complexes based on a fully oxygen-based coordination sphere to the Co(SAL)_2_ complex **3** for OMRP applications. Although the initial results presented here show lower performance in the homopolymerization of VAc and alternating copolymerization of VAc and MAF-TBE relative to the acac complex **1a**, because of less favorable trapping equilibria, the system has potential in the controlled radical polymerization of other LAMs such as fluorinated olefins and work along this direction is currently ongoing in our laboratories.

## Supplementary Materials

The following are available online at www.mdpi.com/2073-4360/9/12/702/s1. Tables of data for bulk VAc homopolymerization and VAc/MAF-TBE alternating copolymerization, stacked plots of the evolution of the ^1^H- and ^19^F-NMR spectra for the copolymerization, and ^1^H-, ^19^F- and ^13^C{^19^F}-NMR spectra of the isolated poly(VAc-*alt*-MAF-TBE) copolymer.
