# Supplementary material for: Bis(formylphenolato)cobalt(II)-Mediated Alternating Radical Copolymerization of tert-Butyl 2-Trifluoromethylacrylate with Vinyl Acetate
Source: Polymers (Basel). 2017 Dec 12;9(12):702. doi: 10.3390/polym9120702 (PMC6418862; doi:10.3390/polym9120702)
Supplement: Supplementary file 1 [file polymers-09-00702-s001.pdf]

polymers

Bis(formylphenolato)cobalt(II)-Mediated Alternating Radical Copolymerization of *tert*-Butyl 2-Trifluoromethylacrylate with Vinyl Acetate

Sanjib Banerjee [1,†], Ekaterina V. Bellan [2], Florence Gayet [2], Antoine Debuigne [3], Christophe Detrembleur [3], Rinaldo Poli [2,4,*], Bruno Améduri [1] and Vincent Ladmiral [1,*]

1 Institut Charles Gerhardt Montpellier, University of Montpellier, CNRS, ENSCM, Place Eugène Bataillon, 34095 Montpellier CEDEX 5, France; sanjib.banerjee@iitbhilai.ac.in (S.B.); bruno.ameduri@enscm.fr (B.A.)
2 Laboratoire de Chimie de Coordination (LCC), Université de Toulouse, CNRS, UPS, INPT, 205 route de Narbonne, BP 44099, 31077 Toulouse CEDEX 4, France; ekaterina.bellan@gmail.com (E.V.B.); florence.gayet@lcc-toulouse.fr (F.G.)
3 Center for Education and Research on Macromolecules (CERM), University of Liege, CESAM Research Unit, Sart-Tilman B6a, 4000 Liege, Belgium; adebuigne@ulg.ac.be (A.D.); christophe.detrembleur@ulg.ac.be (C.D.)
4 Institut Universitaire de France, 1, rue Descartes, 75231 Paris CEDEX 05, France
* Correspondence : rinaldo.poli@lcc-toulouse.fr (R.P.); vincent.ladmiral@enscm.fr (V.L.)
† Present address: Department of Chemistry, Indian Institute of Technology Bhilai, GEC Campus, Sejbahar, Raipur 492015, Chhattisgarh, India.

SUPPORTING INFORMATION

Table S1. Data for bulk VAc polymerization initiated by V-70 in presence or absence of $Co(SAL)_2$ at 30 °C.[a]

Entry	Initiating system	Time (h)	Conversion (%)[b]	$10^{-4} \cdot M_{n,theo.}$ (g mol^{-1})[c]	$10^{-4} \cdot M_{n,SEC}$ (g mol^{-1})[d]	Đ[d]
1	V-70/$Co(SAL)_2$	0.5	0	-	-	-
		1	1	0.07	-	-
		2	5	0.24	-	-
		3	10	0.46	-	-
		4	16	0.72	-	-
		5	22	0.98	3.50	1.62
		20.25	67	2.90	3.87	1.66
2	V-70	0.5	3	n.a.	-	-
		1	7	n.a.	-	-
		2	14	n.a.	-	-
		3	24	n.a.	-	-
		4	32	n.a.	-	-
		5	37	n.a.	4.88	1.60
		21.5	85	n.a.	4.05	1.99

[a]Conditions: $\{[VAc]_0/[V\text{-}70]_0 = 500/2, [V\text{-}70]_0/[Co(SAL)_2]_0 = 2/1$. [b]Determined by ^1H NMR spectroscopy. [c]The theoretical molar masses of the polymers ($M_{n,theo}$) were calculated using the following equation:

$$M_{n,theo.} = \left(\frac{[\mathrm{VAc}]_0}{[\mathrm{Co(SAL)_2}]_0} \times \text{conv. of VAc } \times M_{VAc}\right) + (M_{TEMPO} - 1) + \text{ contribution of V} - 70 \text{ part (S1)}$$

[d]Determined by SEC in THF with multiangle DLS detection and analysis on the basis of the dn/dc.

Table S2. Data for the copolymerization of VAc and MAF-TBE mediated by $Co(SAL)_2$ at 40 °C.[a]

Time (h)	VAc conversion (%/no. of moles)[b]	MAF-TBE conversion (%/no. of moles)[c]	Total conversion (%)	$10^{-3} \cdot M_{n,theo.}$ (g mol^{-1})[d]	$10^{-3} \cdot M_{n,SEC}$ (g mol^{-1})[e]	Đ[e]
2	4/3.4	4/3.1	4	3	17.8	1.75
5	15/12.1	14/11.7	14.5	11.3	28.9	1.65
7	26/20.5	25/20.0	25.5	18.9	42.5	1.61
10	32/26.4	31/25.1	31.5	23.4	48.9	1.59
12	42/33.8	41/33.2	41.5	31.7	57.5	1.58
30	76/61.2	77/62.0	76.5	57.4	83.0	1.55

[a]Conditions: $\{[VAc]_0 + [MAF\text{-}TBE]_0\}/[V\text{-}70]_0/[\mathbf{3}]_0 = 537/3/1$, $[VAc]_0/[MAF\text{-}TBE]_0 = 1/1$, $[V\text{-}70]_0/[Co(SAL)_2]_0 = 3/1$. [b]Determined by ^1H NMR spectroscopy. [c]Assessed by ^{19}F NMR spectroscopy. [d]The theoretical molar mass of the polymers ($M_{n,theo}$) were calculated using the following equation:

$$M_{n,theo.} = \left(\frac{[\mathrm{VAc}]_0}{[\mathrm{Co(SAL)_2}]_0} \times \text{conv. of VAc } \times M_{VAc}\right) + \left(\frac{[\mathrm{MAF-TBE}]_0}{[\mathrm{Co(acac)_2}]_0} \times \text{conv. of MAF} - \text{TBE } \times M_{MAF-TBE}\right)$$
$$+ (M_{TEMPO} - 1) + M_{V70\,fragment} \text{(S2)}$$

With M_{VAc} = 86 gmol^{-1}, $M_{MAF\text{-}TBE}$ = 196 gmol^{-1}, M_{TEMPO} = 155 gmol^{-1} and $M_{V70\,fragment}$ = 141 gmol^{-1}.

[e]Determined by SEC in DMF (containing 0.1 wt% LiCl), system was calibrated using poly(methyl methacrylate) standards.

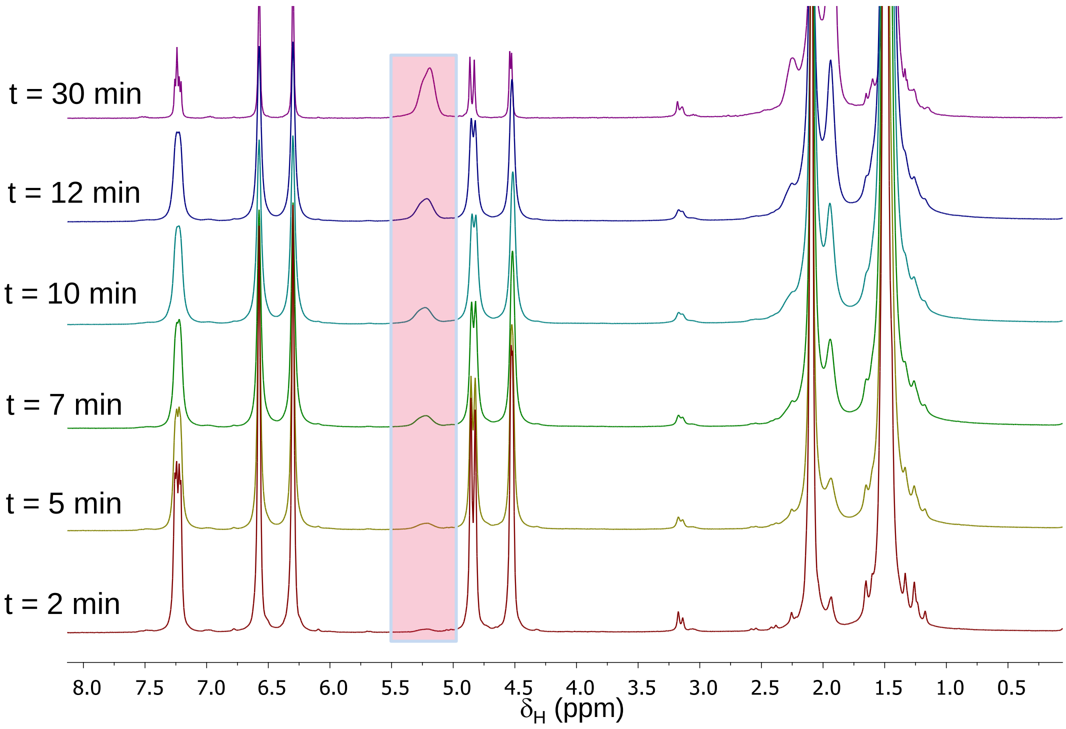

Figure S1. Time evolution of the ^{1}H NMR spectra (in acetone-*d*6, 20 °C) of the bulk copolymerization of VAc and MAF-TBE at 40 °C initiated by V-70 in the presence of $Co(SAL)_2$. $\{[VAc]_0+[MAF\text{-}TBE]_0\}/[V\text{-}70]_0/[\mathbf{3}]_0 = 537/3/1$, $[VAc]_0/[MAF\text{-}TBE]_0 = 1/1$ (Table S2).

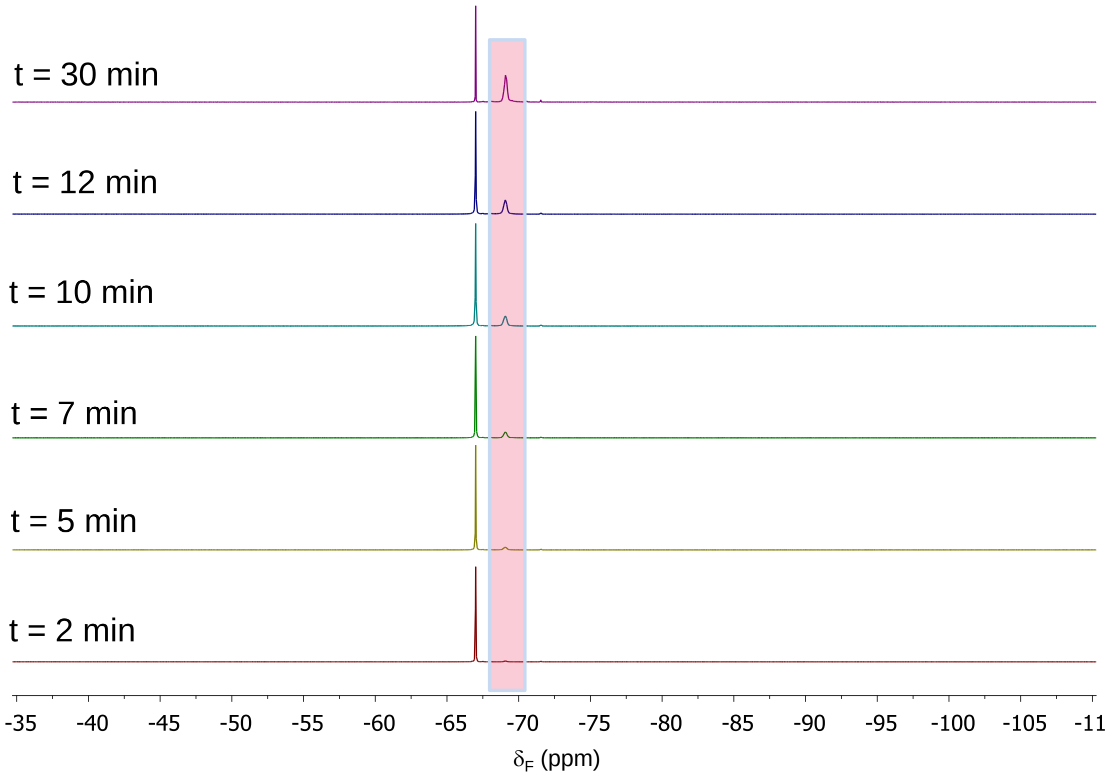

Figure S2. Time evolution of the ^{19}F NMR spectra (acetone-*d6*, 20 °C) during the bulk copolymerization of VAc and MAF-TBE at 40 °C initiated by V-70 in presence of $Co(SAL)_2$ $\{[VAc]_0+[MAF\text{-}TBE]_0\}/[V\text{-}70]_0/[\mathbf{3}]_0 = 537/3/1$, $[VAc]_0/[MAF\text{-}TBE]_0 = 1/1$ (Table S2).

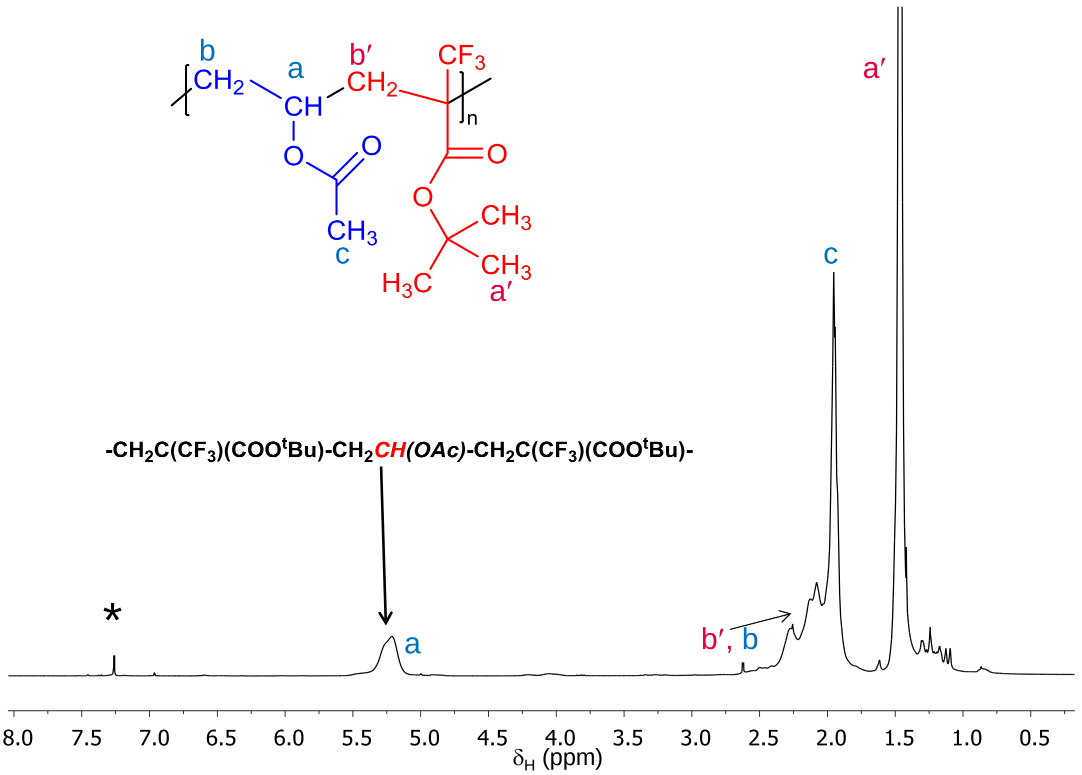

Figure S3. Representative^{1}H NMR spectrum (400 MHz) of poly(VAc-*alt*-MAF-TBE) copolymer prepared by OMRP of VAc with MAF-TBE mediated by V-70/Co(SAL)$_2$ at 40 °C in bulk (Table S2), recorded in $CDCl_3$ at 20 °C. Peaks are centered at δ 1.45 (a′), 1.90 (c), 2.05 to 2.75 (b and b′), 5.20 (a). (*) Solvent ($CHCl_3$) peak.

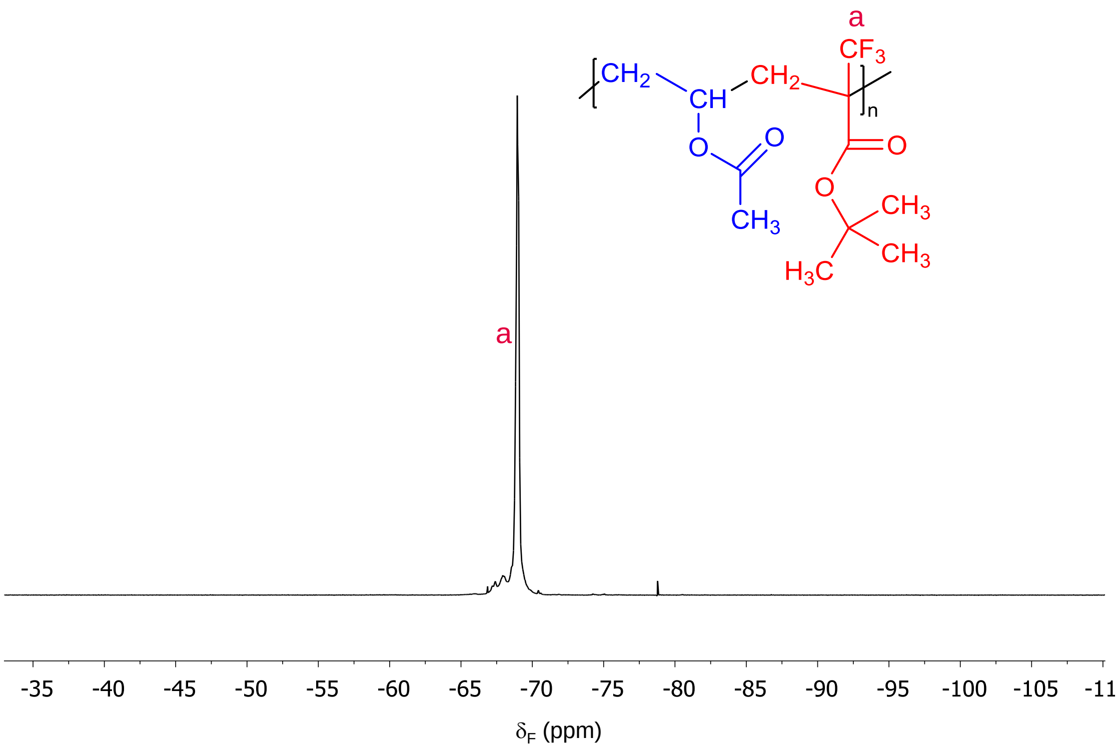

Figure S4. Representative ^{19}F NMR (376 MHz) spectrum of poly(VAc-*alt*-MAF-TBE) copolymer prepared by OMRP of VAc with MAF-TBE mediated by V-70/Co(SAL)$_2$ at 40 °C in bulk (Table S2), recorded in $CDCl_3$ at 20 °C. The peak is centered at -69 ppm.

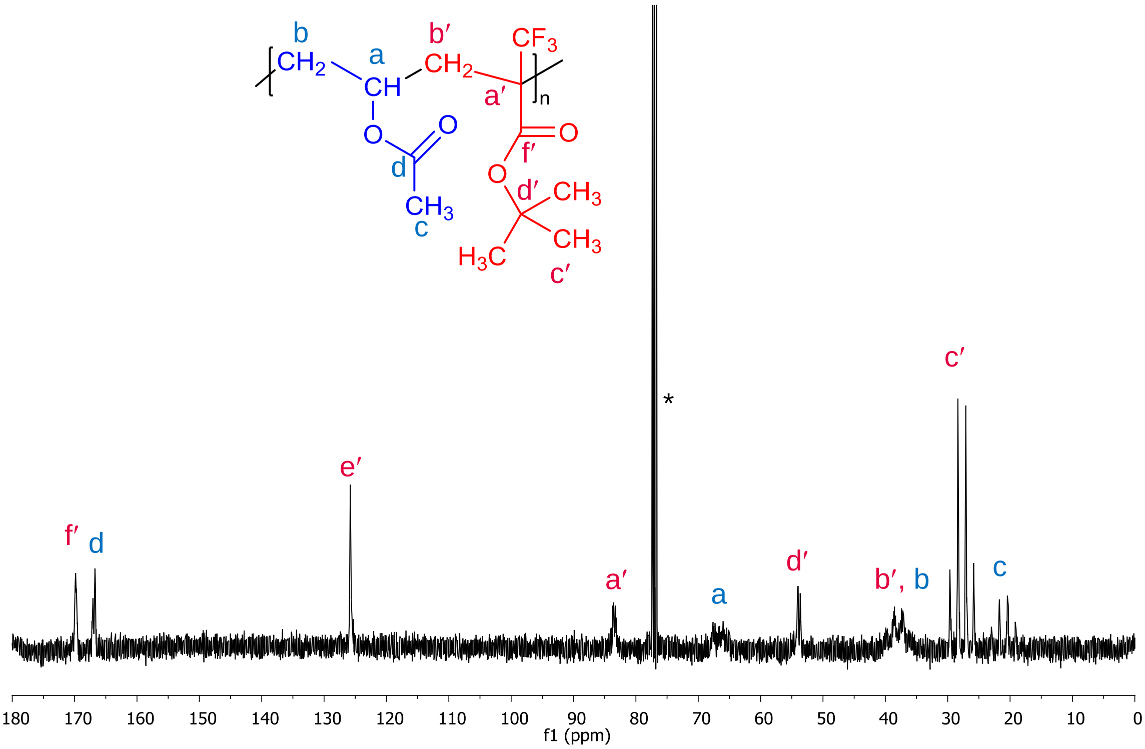

Figure S5. Representative $^{13}C\{^{19}F\}$ NMR spectrum (100 MHz) of poly(VAc-*alt*-MAF-TBE) copolymer prepared by OMRP of VAc with MAF-TBE mediated by V-70/Co(SAL)$_2$ at 40 °C in bulk (Table S2), recorded in $CDCl_3$ at 20 °C. Peaks are centered at δ 20.8 (c); 27.8 (c′), 38.4 (b and b′), 53.5 (d′), 67.5 (a), 83.2 (a′), 125.8 (e′), 167.0 (d), and 170.0 (f′). (*) $CDCl_3$ peak.
